# The emerging role of MicroRNA-182 in tumorigenesis; a promising therapeutic target

**DOI:** 10.1186/s12935-023-02972-0

**Published:** 2023-07-12

**Authors:** Pouriya Sameti, Maryam Tohidast, Mohammad Amini, Seyedeh Zahra Bahojb Mahdavi, Souzan Najafi, Ahad Mokhtarzadeh

**Affiliations:** Immunology Research Center, Tabriz University of Medical Sciences, Tabriz, Iran

**Keywords:** MicroRNA-182, Cancer, Signaling pathways

## Abstract

A wide range of studies have indicated that microRNAs (miRNAs), a type of small single-stranded regulatory RNAs, are dysregulated in a different variety of human cancers. Therefore, they are expected to play important roles in tumorigenesis by functioning as oncogenic (oncomiRs) or tumor-suppressive miRNAs. Subsequently, their potential as diagnostic and therapeutic targets for malignancies has attracted attention in recent years. In particular, studies have revealed the aberrant expression of miR-182 through tumorigenesis and its important roles in various aspects of malignancies, including proliferation, metastasis, and chemoresistance. Accumulating reports have illustrated that miR-182, as a dual-role regulator, directly or indirectly regulates the expression of a wide range of genes and modulates the activity of various signaling pathways involved in tumor progression, such as JAK / STAT3, Wnt / β-catenin, TGF-β, and P13K / AKT. Therefore, considering the high therapeutic and diagnostic potential of miR-182, this review aims to point out the effects of miR-182 dysregulation on the signaling pathways involved in tumorigenesis.

## Introduction

Although the global Covid-19 pandemic has attracted worldwide attention in recent years, cancer remains one of the diseases that has endangered lives for many years, and it puts a lot of costs on the people and the government every year. Hence, the international community is looking for potential treatments for this costly disease. Cancer is the consequence of the out-of-control division of cells that can be caused by mutations in genes that control cell division and cell death. In other words, it is the result of the imbalance between cell division and death [1]. Cancer hallmarks have been identified in several studies and experiments to better understand the behavior of malignant cells. These hallmarks include growth signal independency, insensitivity to anti-growth signals, continuous angiogenesis, limitless cell proliferation, invasion, evading apoptosis, metastasis, mutation, evading immune response, and changeability of genome [2]. In line with public awareness, GLOBOCAN in 2018 published statistics on the incidence and mortality of various cancers. It showed 18.1 million new cases of cancer and 9.6 million cancer-related deaths in 2018. Among them, lung cancer ranks first in terms of incidence and mortality rate in both sexes, male and female. A closer look at these statistics shows that breast cancer, prostate cancer, and colorectal cancer (CRC) have the highest prevalence after lung cancer, respectively. And also, CRC, stomach cancer, and liver cancer are the second to fourth deadliest malignancies after lung cancer, respectively [3]. With the increase in the incidence of cancer in different societies, treatment methods for this disease are developing day by day. Common therapeutic options include surgery, chemotherapy, hormonal therapy, radiation therapy, and immunotherapy [4]. Among these, chemotherapy combined with surgery is one of the most well-known methods. For all its benefits, chemotherapy has its drawbacks, of which multi-drug resistance (MDR) is the most important one. This problem is mainly defined as the resistance of cancer cells to a drug used in chemotherapy and at the same time resistance to other chemotherapies. MDR can be explained by various mechanisms, such as cancer stem cells, epigenetic regulation, resistance to apoptosis, DNA damage and repair, and microRNAs [5]. If we want to take a brief look, 1999 can be considered the year of RNA interference (RNAi) discovery and a revolution in RNA regulation. These regulatory RNAs, including microRNAs and siRNAs, demonstrated their function in silencing target genes and became a new therapeutic strategy against cancer cells [6]. In recent years, microRNAs have attracted the attention of expert researchers and scientists in this field, both as a mechanism involved in multidrug resistance in the chemotherapy process and as a new strategy with strong potential in the treatment of various cancers. When it comes to regulating genes in the post-transcriptional phase, microRNAs play the main role [7]. MicroRNAs, small non-coding regulatory RNAs with a length of 18–22 nucleotides, are classified in highly conserved genes responsible for regulating gene expression by inhibiting translation mostly through binding to 3’UTR of target mRNA. It should be noted that RNA POL II also is responsible for the transcription of these microRNAs [5, 8, 9]. A growing body of studies has highlighted the importance of microRNAs in cancer initiation and progression, indicating their diagnostic and therapeutic significance [10, 11]. MicroRNAs may either function as tumor suppressors or as oncogenes in various malignancies by regulating multiple target genes. However, due to the diversity of their target gene, some microRNAs have been found to play a dual role in tumorigenesis [7, 12]. Subsequently, in this review, we intend to discuss the significance of a dual-role microRNA, namely miR-182, in tumorigenesis to give insights into its potential as a promising diagnostic and therapeutic target in various cancers.

## MicroRNA biogenesis and mechanism of action

Addressing the biogenesis of microRNAs can give us a better understanding of how these regulatory RNAs are processed and involved in regulating gene expression. More than two-thirds of microRNAs are expressed from specific genes, and less than one-third are generated through the intron processing of protein-coding genes. The transcription of these genes is mostly carried out by RNA polymerase II enzyme (RNA Pol II). When RNA Pol II is activated, the primary transcript of these genes, called primary microRNA (pri-miRNA), is formed like mRNAs, having a 7-methylguanosine cap and a poly-A tail, but with the difference that it has a hairpin structure. Continuation of miRNA processing occurs with the activity of RNase III enzyme Drosha and DGCR8 RNA-binding protein, together known as the Microprocessor complex, cleaving pri-miRNA to pre-miRNA. The pre-miRNA has a stem-loop structure and about 60–70 nucleotide length. The cytoplasm is the next host for the continuation of pre-miRNA processing, and Exp5 / RanGTP is responsible for this transfer from the nucleus to the cytoplasm. After this transfer, and just before Dicer begins to act as an RNase to cleave pre-miRNA and convert it to miRNA duplex intermediate, RanGAP releases pre-miRNA from Exportin5 by consuming a phosphate and converting GTP to GDP. For this immature double-stranded miRNA to achieve its function of gene destroying and silencing, the argonaute (Ago) protein binds to the duplex to separate one of the pre-miRNA strands, leaving a strand attached to the Ago protein. The separated strand is called the passenger strand, while the remaining strand with Ago is known as the mature miRNA which after this formation and entry into the RISC complex, they are ready to control gene expression [13–15]. PAZ and MID are the two major domains of Ago proteins, which bind to the 3’ and 5’ UTR of target mRNA, respectively. In addition, studies have shown that the 7-methyl guanine cap, the binding site of translation factors, such as eIF4E, is blocked by the mammalian Ago2 protein to prevent the translation process from starting [16–18]. GW182 is considered a scaffold molecule that interacts with argonaute proteins to suppress the translation and degradation of target mRNAs and thus suppress gene expression. It is noteworthy that this interaction leads to deadenylation and decapping of target mRNAs by mRNA adenylase and DCP2, respectively, which is complemented by the nuclease effect of the XRN1 exonuclease enzyme [19].

## MicroRNA-182

Studies indicated that microRNAs play significant roles in molecular and biological processes, and their dysregulation may be involved not only in tumorigenesis but also in other diseases. miR-182 is a well-studied microRNA located on chromosome 7q31-34 that belongs to the miR-183 family. miR-182 is no exception, and it is found to participate in various diseases as well [20]. For example, studies on alcoholic hepatitis (AH), which is more common in alcoholics with underlying alcoholic liver disease (ALD), suggests the role of miR-182 in elevating the expression of inflammatory mediators, leading to increased liver cell damage in patients [21]. Other studies focusing on autoimmune diseases have shown that IFN-γ, which induces CD4^+^ Th1 and inflammation in Relapsing-remitting MS patients, is upregulated following miR182 overexpression [22]. In addition, other studies indicated that miR-182-5p has protective effects on liver ischemia-reperfusion and cerebral ischemia-reperfusion injuries. miR-182-5p inhibits the release of proinflammatory cytokines, such as IL-6 and TNF-α, by targeting TLR4 (toll-like receptor 4), an intrinsic immune signaling receptor, to exert a protective effect on the side effects of liver and cerebral ischemia-reperfusion injuries, such as suppression of the inflammatory response [23, 24]. Overall, miR-182 can be considered an important microRNA in the normal functioning of human cells.

## MicroRNA-182 in human cancers

**Fig. 1:**
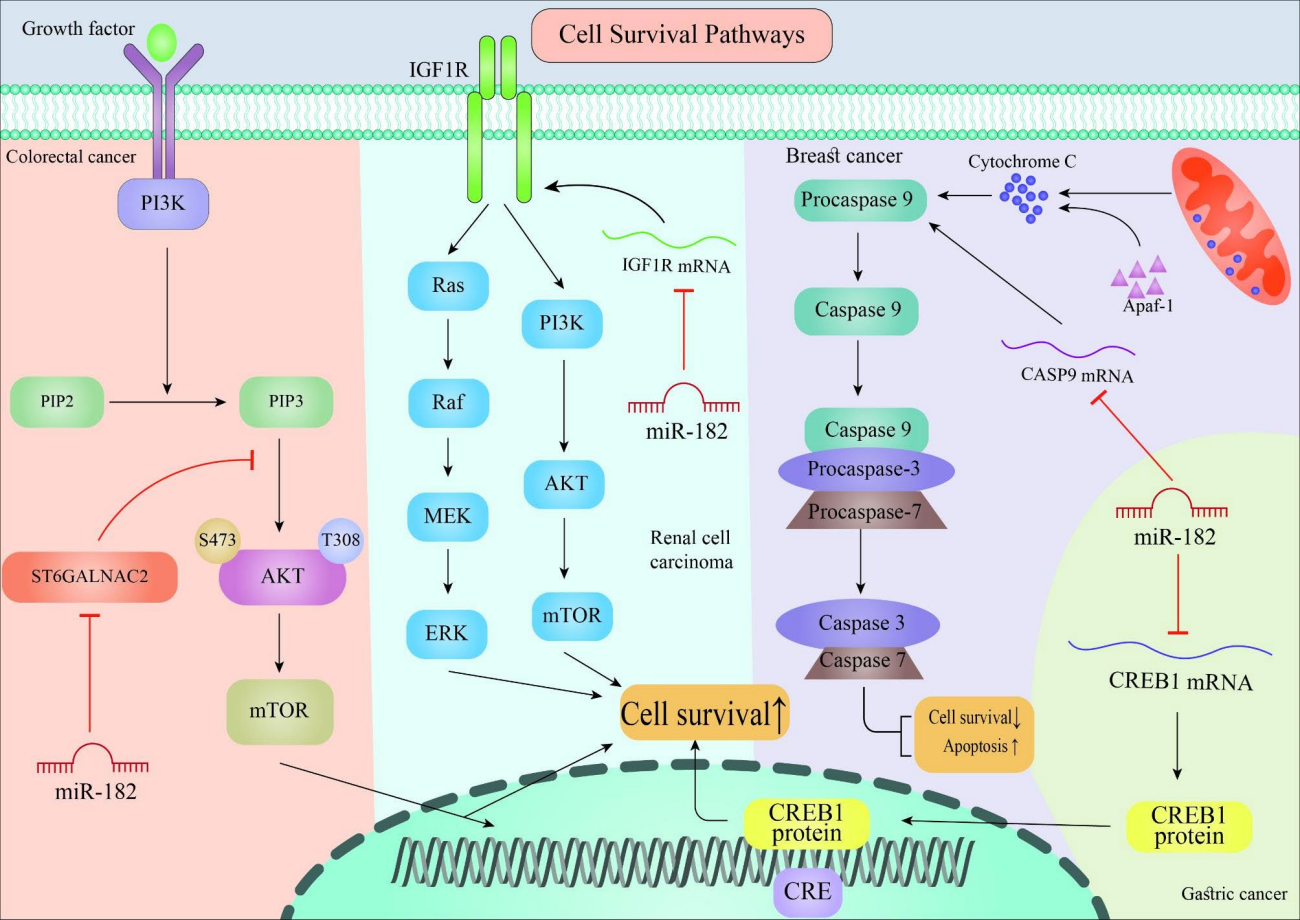
The schematic view of signaling pathways involved in cell survival in various human cancers, including renal cell carcinoma, and colorectal, breast, and gastric cancers. As represented, miR-182 plays a dual role in regulating cancer cell survival

Extensive studies conducted on different microRNAs indicate their role in tumorigenesis, and miR-182, a member of this large family of regulatory RNAs, is no exception. Also, studies showed that miR-182 is dysregulated in various types of cancer. A growing body of studies has established that miR-182, depending on the type of cancer and the signaling pathways involved, can act as both a tumor suppressor and an oncogene to modulate tumorigenesis, indicating the dual role of this microRNA in human cancers [25–27]. For example, various studies have shown that miR-182 can either suppresses or inhibits the survival of malignant cells by regulating different signaling pathway, as depicted in Fig. 1. In the following sections, we will have a closer look at the role of miR-182 in the progression of different cancer types.

### Gastric cancer

Gastric cancer (GC) is considered one of the most common malignant tumors of the gastrointestinal tract [28, 29]. The majority of GC cases are adenocarcinomas. According to the Cancer Genome Atlas (TCGA), GC can be classified into four molecular subgroups: genomically stable (GS), chromosomal instability (CIN), microsatellite instability (MSI), and EBV-positive (EBV) [30]. Regarding global statistics, although this cancer is in fifth place in terms of prevalence, GC remains a deadly malignancy, ranking third in terms of mortality [3]. Although surgery in combination with chemotherapy and radiotherapy has made substantial improvements in GC treatment [31], poor prognosis in the early stages leads to tumor growth and systemic metastasis in patients, failing the treatment [31, 32]. GC is a multi-step and multi-factorial process [33] that is influenced by multiple environmental factors, such as Helicobacter pylori infection, and also genetic factors, such as DNA methylation, gene deletion and amplification, epigenetic inactivation of several genes, and aberrant somatic mutations [34, 35]. Numerous studies have been performed on the molecular mechanisms and association between microRNAs and tumorigenesis to formulate a potential treatment plan for GC. It has been reported that several microRNAs, including miR-182, can act as a tumor suppressor or oncogene in this cancer and affect various cellular processes, such as migration, cell proliferation, apoptosis, and invasion [36, 37].

Studies in this field have shown that expression of miR-182 was higher in GC tissues compared to normal adjacent tissues. Also, the expression of this microRNA in HGC-27 cell line was significantly higher than that of other GC cell lines. In contrast, RAB27A mRNA and protein levels were evidenced to be lower in GC tissues than in adjacent tumor tissues, showing a reverse correlation with miR-182 expression [36]. RAB27A is a member of the small GTPase family [38] and suppresses cell motility and invasion in various human cancers as a tumor suppressor [39, 40]. It was illustrated that targeting miR-182-5p could induce apoptosis and cell cycle arrest at G0/G1 stage, as well as reduce cell viability, invasion, and migration by upregulating RAB27A [36].

Opposite to the previous study, a study indicated that miR-182 was downregulated through gastric tumorigenesis, illustrating tumor suppressive function. After transferring miR-182 antisense oligonucleotide and pri-miR-182 into the gastric adenocarcinoma cells, it was observed that increased expression of miR-182 suppresses the growth of gastric adenocarcinoma. According to this report, miR-182 directly targets the 3’-UTR of CREB1 mRNA and negatively regulates CREB1 mRNA and protein expression in gastric adenocarcinoma [37]. The cAMP-responsive element binding protein 1 (CREB1) is a DNA-binding protein belonging to the leucine zipper transcription factor family [41]. CREB1 oncogene is capable of regulating several microRNAs in various cancers, such as GC, and is involved in tumor growth and cell proliferation [37, 42]. Subsequently, as depicted in Fig. 1, it can be stated that miR-182 can also function as a tumor suppressor and inhibit GC progression by regulating CREB1 activity [37].

Another study also highlighted the tumor suppressive role of miR-182, showing a negative feedback loop between ANUBL1 and miR-182. Downregulation of miR-182 expression in GC tissue was also reported in this study. In contrast, qRT-PCR, western blot, and immunohistochemistry analyses evidenced that ANUBL1 expression in GC tissues was higher than that of normal tissues, and ANUBL1 may act as an oncogene. Furthermore, ANUBL1 overexpression was shown to promote colony formation and proliferation in SGC-7901 cells. ANUBL1, also known as ZFAND4 (zinc finger, AN1-type domain 4), has been shown to suppress anti-proliferative microRNAs, including miR-182, contributing to GC progression. On the other hand, miR-182 was also found to negatively regulate the expression of this gene by targeting the 3’-UTR of ANUBL1 mRNA. Also, it was reported that ANUBL1 cDNA lacking the predicted 3’-UTR sites abrogates miR-182 cellular function [43].

In the other related study, miR-182 has been found to inhibit gastric tumorigenesis by targeting target HOXA9 [44]. The homeobox (HOX) proteins are transcription factors that play a significant role in cell development and differentiation by interacting with various types of proteins. They, moreover, can act as oncogenes or tumor suppressors in a different variety of human cancers [45]. It was shown that miR-182 overexpression or HOXA9 suppression was able to hamper the ability of GC cells to proliferate, invade, and migrate. On the other hand, it was evidenced that RUNX3 (RUNX Family Transcription Factor 3) can regulate the expression of miR-182 by directly binding to its promoter and inducing its expression [44]. RUNX3 transcription factor is epigenetically inactivated through gene silencing or protein mislocalization in GC [46]. Overall, RUNX3-mediated upregulation of miR-182 can inhibit GC cell migration, invasion, and proliferation in vitro and tumor growth in vivo by modulating HOXA9 expression. These findings indicated the therapeutic value of targeting the RUNX3/miR-182/HOXA9 axis for GC [44].

Although Cisplatin (CDDP) is considered an essential component of chemotherapy for the treatment of GC, unfortunately, after several periods of use, the resistance of GC cells to this drug limits the chemotherapy process [47]. Studies have shown that Circular RNAs (Circ RNAs) act as tumor suppressors or oncogenes in cancer pathogenesis and regulate several processes in tissues and cells [48, 49]. Interestingly, increased expression of CircRNAs is an important reason for CDDP resistance in GC cells. Also, CircRNAs can inhibit the function of microRNAs by acting as competitive endogenous RNAs (ceRNAs) and “sponging” microRNAs. A study based on the RNA FISH assays, has shown a correlation between cytoplasmic expression of Circ-FN1 and miR-182-5p. Briefly, Circ-FN1 acts as a ceRNA via sponging miR‐182‐5p and eliminates the ability of this microRNA to activate apoptosis in GC cells. Researchers showed that circFN1 expression levels were higher in CDDP-resistant tissues and cell lines, and according to the findings, increased Circ-FN1 expression level was significantly associated with aggressive biological behaviors. Studies have shown that there is a relationship between miR-182-5p expression level and CDDP resistance in GC tissues, and miR-182-5p was downregulated in CDDP-resistant GC tissues. Furthermore, based on this study, the downregulation of Circ-FN1 increased CDDP chemosensitivity of GC cells in vivo and in vitro by targeting miR‐182‐5p [50].

In another study, researchers showed that the expression level of Circ-SFMBT2, as a biomarker for cancer diagnosis, was higher in plasma and tissue samples of GC patients. The luciferase assay evidenced that Circ-SFMBT2 acts as a sponge for miR-182-5p, and upregulates CREB1 mRNA expression, thereby leading to GC cell proliferation. The transfection of GC cells with Circ-SFMBT2 siRNA and miR-182-5p mimics reduced CREB1 mRNA and protein expression and diminished the ability of GC cells to proliferate. These results confirmed that overexpression of Circ-SFMBT2 leads to abnormal expression of CREB1 by downregulating miR-182-5p. On the other hand, it was reported that miR-182-5p inhibited cell proliferation in GC by directly targeting the 3′-UTR of CREB1 mRNA [51]. A recent study has shown that Circ-0001658, another member of CircRNAs, is dysregulated in GC, and its overexpression induces the expression of RAB10 oncogene by sponging miR-182. The knockdown of this circular RNA increased miR-182 expression and promoted miR-182-mediated RAB10 downregulation, thereby inducing apoptosis and inhibiting GC cell viability and autophagy. As a member of the Ras protein family, RAB10 is a direct target of miR-182, which plays a key role in regulating autophagy with its oncogenic function in various cancers [52].

Overall, it can be concluded that miR-182 mainly exerts tumor-suppressive effects in GC cells, and considering its involvement in chemosensitivity, miR-182 possesses great potential as a therapeutic target for this malignancy.

### Hepatocellular carcinoma

**Fig. 2:**
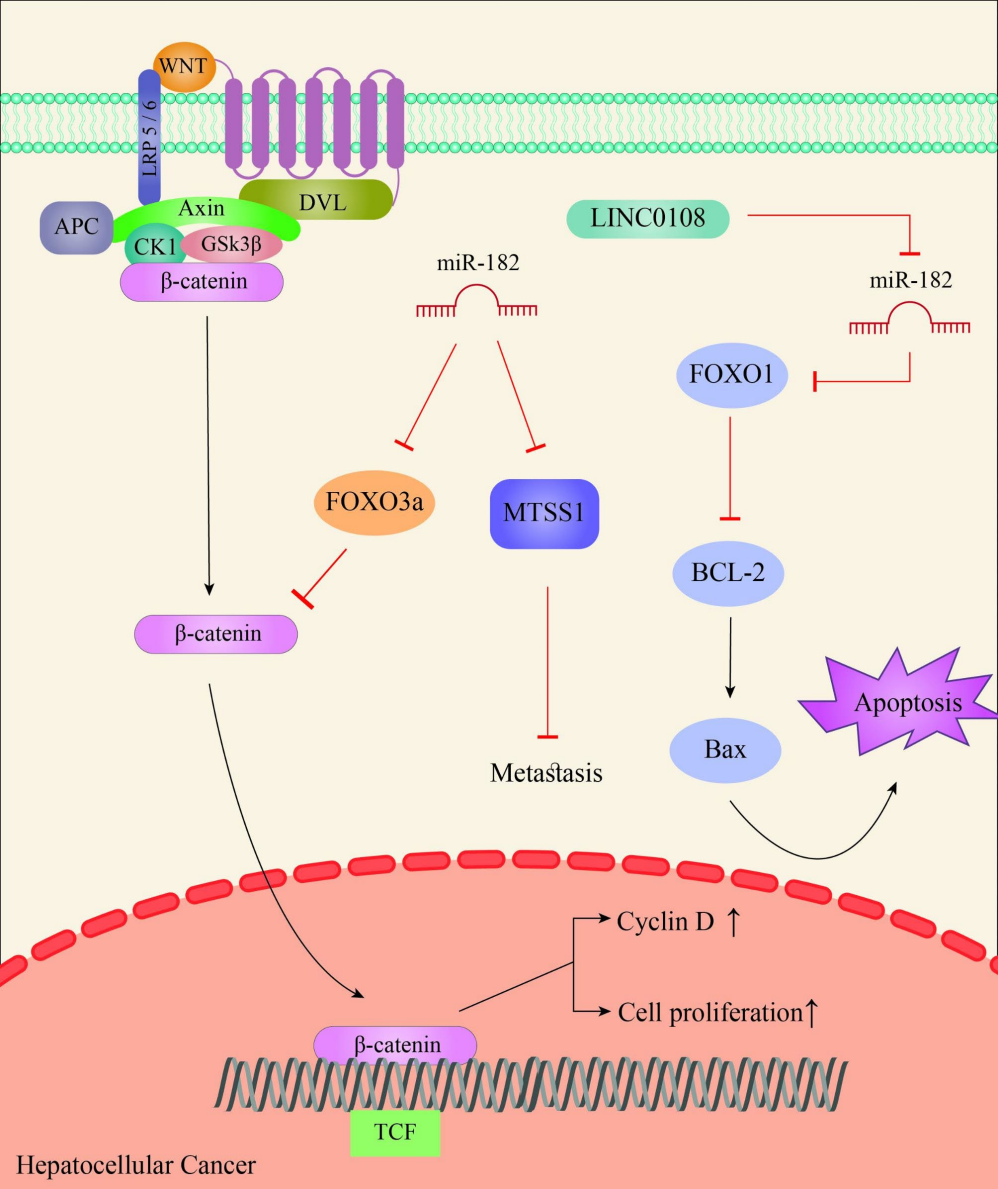
Schematic representation of hepatocellular carcinoma indicates that FOXO3a, an inhibitor of β-catenin/TCF pathway, is suppressed by miR-182, leading to cell proliferation. On the other hand, miR-182 targets FOXO1, an apoptosis inducer, and suppresses cell apoptosis, which can be reversed by LINC0108 expression

Hepatocellular carcinoma (HCC), as the most common primary liver cancer, is one of the most well-known and life-threatening solid cancers in the world, ranking third in terms of mortality rate, with about six hundred thousand deaths annually. In this regard, the clinical community considers liver cirrhosis and viral hepatitis B and C to be determinants of HCC incidence in patients [53–55]. Surgery and liver transplantation, followed by chemotherapy, are considered the main strategies to combat HCC. However, for some reasons, such as metastasis, recurrence, and drug resistance, the disease faces a poor prognosis which reduces the overall 5-year survival of patients to less than 5% [56–58]. Therefore, the existence and highlighting of new molecular targets can be a bright light in this darkness. Extensive studies on the role of miR-182 in HCC have shown that this microRNA often has an oncogenic function, and it is upregulated in this cancer. Regarding HCC cell metastasis, studies indicated that Metastasis suppressor 1 (MTSS1), a gene involved in regulating cytoskeletal motility, is important in inhibiting HCC cell metastasis and has been identified as the target gene for miR-182. Overexpression of miR-182 enhanced HCC metastasis by targeting MTSS1, so research has shown that targeting miR-182 has the opposite effect and enhanced the anti-metastatic properties of MTSS1 (Fig. 2) [57]. Several studies investigating the behavior of miR-182 in HCC have shown that this regulatory RNA particularly targets members of the forkhead box transcription factor family. For example, miR-182 downregulated FOXO3a by targeting 3’UTR of FOXO3a to promote the Wnt/β-catenin signaling pathway. FOXO3a binds to β-catenin in competition with TCF and prevents the transcription of downstream genes involved in the cell cycle, such as CyclinD. miR-182 inhibits the function of this tumor suppressor gene and also increases the interaction between TCF/β-catenin to activate genes involved in the cell cycle, which ultimately leads to HCC cell proliferation (Fig. 2). Other studies have shown that miR-182-5p increases cell proliferation and decreases HCC apoptosis by targeting FOXO1, another member of the forkhead box family, 1. FOXO1 has tumor-suppressive properties and induces apoptosis by suppressing Bcl-2 and promoting Bax activity. The results showed that FOXO1 is downregulated by miR-182-5p overexpression. It was also shown that LINC0108 upregulation, a member of the long noncoding RNAs, increased FOXO1 expression followed by enhancement of the tumor suppressive effects of FOXO1 in HCC cells by sponging miR-182-5p (Fig. 2) [56, 59]. Because the role of chemotherapy in the treatment of cancers, including HCC, is important, the existence of problems, such as drug resistance, are strong barriers to this type of treatment. Cisplatin is a chemotherapy drug used in HCC treatment. Studies focusing on the effects of miR-182 in HepG2 cell drug resistance to Cisplatin have shown that miR-182 is overexpressed in this type of HCC cell. It was evidenced that miR-182 partially induced HCC cell cisplatin resistance by targeting a proapoptotic gene, called TP53INP1, which plays an important role in cell cycle arrest and induction of apoptosis [58]. Besides, the majority of studies have confirmed the oncogenic activity of miR-182 in HCC. PDCD4, a gene involved in tumor suppression by inhibiting the helicase activity of eIF4A and protein translation, is a direct target for miR-182 and is downregulated by overexpression of miR-182 [60]. Ephrin5A, a negative regulator of EGFR signaling, acts as a tumor suppressor in HCC and is targeted by miR-182. The results of the experiments showed a decrease in HCC cell proliferation following the suppression of miR-182 expression and upregulation of Ephrin5A [61]. Solid tumors, such as HCC, are mainly hypoxic due to the rapid growth and low capillary density, so angiogenesis in these tumors to resolve the oxygen need is common. RASA1 has been identified as an important anti-angiogenic factor in HCC, which happened to be downregulated in this malignancy. The results of studies on this issue indicated that the downregulation of RASA1 is directly related to the overexpression of miR-182, thus it was suggested that miR-182 can promote HCC cell angiogenic ability by targeting RASA1 [62].

### Colorectal cancer

A global statistical survey has revealed that CRC is ranked third and second in terms of prevalence and mortality, respectively [63]. Genetic and epigenetic alterations are two important factors in the multistage process of CRC development from normal epithelial cells to malignant carcinoma [64, 65]. Despite advances in CRC treatment strategies, poor prognosis, metastasis, and drug resistance continue to be the main reasons for the low survival of patients and cancer deaths [66, 67]. As previously mentioned, the discovery of new molecular biomarkers is critical for improving the clinical outcomes of CRC patients [68]. In particular, it has been demonstrated that miR-182 plays a key role in CRC tumorigenesis. Numerous studies have reported that miR-182 expression is upregulated in CRC tissues compared to adjacent non-cancerous tissues [69], and its upregulation was associated with positive regional lymph node status, high depth of tumor invasion, and advanced stage of TNM [70]. These findings suggested that a higher level of miR-182 expression considerably promoted CRC invasion, migration, and cell proliferation in vivo and in vitro [66]. In contrast, it has been shown that miR-182-5p expression was dramatically downregulated in both CRC tissues and cell lines, and its overexpression significantly inhibited the invasion, migration, and proliferation of CRC cells in vitro [71].

Lnc-AGER-1, by binding to miR-182 and then sponging this microRNA, could indirectly modulate the expression of the AGER gene in CRC. Lnc-AGER-1 expression is downregulated in CRC tissues relative to adjacent noncancerous tissues, and this decreased expression was associated with clinical T status. Increased expression of lnc-AGER-1 leads to inhibition of proliferation, migration, increased apoptosis, and cell cycle arrest at the G0/G1 phase. The expression of advanced glycosylation end-product specific receptor (AGER) is significantly lower in the colorectal tumor epithelia compared to paired normal mucosa. On the other hand, it has been shown that miR-182 expression is high in CRC and acts as an oncogene. Therefore, it can be inferred that lnc-AGER-1 serves as a ceRNA via sponging miR-182 and regulating AGER [72].

**Fig. 3:**
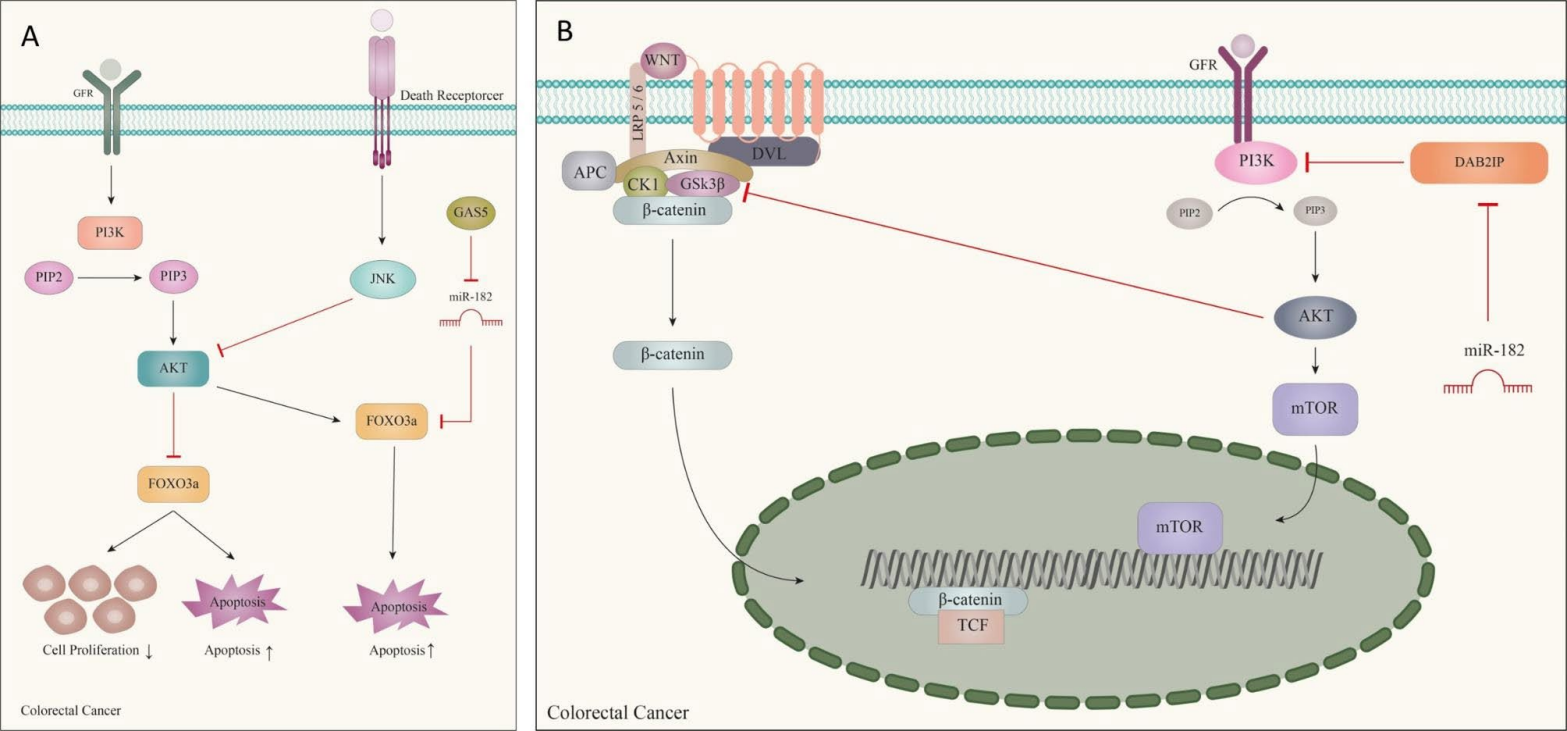
The involvement of miR-182 in the regulation of signaling pathways in colorectal cancer. **(A)** FOXO3a with function of inducing the pathway leading to apoptosis and regulating the cell cycle, is suppressed by miR-182 in colorectal cancer. However, lncRNA GAS5 plays a tumor suppressor role with sponging of miR-182. **(B)** DAB2IP tumor suppressor, which inhibits P13K/AKT/mTOR pathway, is suppressed by the oncogenic functioning of miR-182, leading to CRC cell proliferation

In line with investigating the inverse relationship between GAS5 expression and miR-182‑5p, decreased expression of lncRNA GAS5 has been reported in CRC tissues and cell lines. GAS5 downregulation was significantly associated with lymph node metastasis and advanced clinical stage in CRC [73]. Growth arrest‑specific transcript 5 (GAS5) acts as a momentous regulator of CRC initiation and progression by regulating inflammatory cytokines via NF-κB and Erk1/2 pathways [74]. GAS5 acts as a miR-182‑5p sponge, prevents cell proliferation, and enhances apoptosis in vitro and in vivo in CRC by positively regulating FOXO3a expression [73]. FOXO3a is reported to regulate pro‑apoptotic genes and cell cycle by its tumor-suppressive function in various cancers. FOXO3a is an important target in the PI3K / AKT signaling pathway and may be a direct target of miR-182‑5p [73, 75]. Overall, it can be concluded that the GAS5/miR‑182‑5p/FOXO3a axis can be a potential therapeutic target in CRC (Fig. 3a) [73].

In the same direction as confirming the effects of miR-182 in modulating malignant phenotypes of CRC cells, it was shown that increased invasion, cell proliferation, colony formation, and migration are predictable results of miR-182 overexpression in CRC cells in vitro and in vivo. Also, by examining further mechanisms, it was shown that following the increase in miR-182 expression, mesenchymal marker of Vimentin and Snail expression levels increased, and epithelial marker of E-cadherin and SATB2 expression levels were reduced in vitro and in vivo, causing epithelial-mesenchymal transition (EMT). miR-182 with direct targeting of 3’UTR of SATB2 mRNA suppresses the expression of SATB2 mRNA and protein metastatic CRC tissues and cell lines. Restoring SATB2 expression could reverse the effects of miR-182 on CRC cell proliferation and migration [76]. A member of the nuclear matrix binding protein family called the Special AT-rich sequence binding protein 2 (SATB2), by having a binding site to AT-rich DNA elements of nuclear matrix-attachment regions (MARs), can play its function through changing the structure of chromatin by interacting with metastasis-associated protein 2 (MAT2) and histone deacetylase 1 (HDAC1). As a result, its undeniable role in integrating epigenetic and genetic signals to modulate the transcriptional activity of the gene is highlighted [77], and it induces epithelial-mesenchymal transition via inducing Snail transcription. This negative correlation also indicates that miR-182 might be a pivotal regulator in CRC metastasis [76].

In line with the evaluation of the significance of the miR-182/DAB2IP axis, it was shown that miR-182 is upregulated in CRC tissue and serum samples. Following the increase of miR-182 expression, the level of ki67 protein (a biomarker of proliferation), level of MMP-2 and MMP-9 (invasion markers), and colony formation in CRC were promoted. Mechanistically, miR-182 exerts this oncogenic role in CRC by targeting DAB2IP mRNA and activating the Wnt/β- catenin and PI3K/Akt/mTOR pathways [78]. Downregulation of DAB2IP (DOC-2/DAB2 Interacting Protein) is mainly because of the altered epigenetic regulation of its promoter, which may lead to augmented DNA repairability, radiation resistance, and reduced apoptosis after radiation [79]. As one of the members of the Ras GTPase-Activating Proteins (RAS-GAP) family, DAB2IP functions as a signaling adapter that negatively modulates oncogenic pathways, and plays an important role in epithelial-mesenchymal transition (EMT), cancer cell growth, metastasis, and invasion, during CRC progression. The stimulation of the onset and progression of CRC is often mediated by the Wnt/β-catenin pathway, and control of malignant phenotypes, including angiogenesis, proliferation, and invasion in a variety of cancers, including CRC, is the responsibility of the PI3K / AKT / mTOR pathway (Fig. 3b). These pathways are generally identified as potential targets for CRC prevention. It has also been shown that decreased expression of miR-182, followed by increased DAB2IP expression, inhibited CRC tumor growth in vivo [78].

Forkhead box F2 (FoxF2), as a key member of the forkhead box transcription factors, is downregulated through colorectal tumorigenesis and participates in epithelial-mesenchymal interaction, embryonic development, and extracellular matrix synthesis (ECM). miR-182, by targeting the 3’-UTR of FoxF2 mRNA, inhibits FoxF2 expression and increases β-catenin activity and therefore causes CRC development. Recent research reported that FoxF2 low expression has been linked with several tumorigenic processes, including proliferation, metastasis, and invasion. Increased expression of Wnt5a and activation of the canonical Wnt signaling pathway, which results in tissue decomposition and epithelial depolarization, are among the consequences of FOXF2 inhibition. It was further observed that β-catenin expression and consequently CRC cell invasion and cell growth were inhibited following FOXF2 restoration. FoxF2 has been reported to decrease intestinal adenoma formation, by antagonizing the activity of the Wnt/β-catenin pathway via up-regulating the SFRP1, a suppressor of Wnt signaling. In addition, RECK, an antagonist of matrix metalloproteinases (MMPs), was shown to be repressed by miR-182 overexpression, while tissue inhibitor of metalloproteinase 3 (TIMP3) expression was upregulated by FOXF2 restoration. Furthermore, the effect of the miR-182/FoxF2 axis on ECM readjustment to facilitate metastasis and invasion was confirmed to be exerted by this mechanism [80].

Opposite to previous studies, another study in this field showed that miR-182-5p expression was considerably decreased in both CRC tissues and cell lines. After increasing miR-182-5p expression in SW620 cells, the expression of the epithelial marker E-cadherin increased significantly, and in contrast, the protein expression of mesenchymal marker Vimentin was significantly reduced. miR-182-5p overexpression was able to inhibit MTDH protein expression by binding to the 3’-UTR of MTDH mRNA, thereby preventing invasion, proliferation, and metastasis in CRC cells. It has also been reported that some biological behaviors involved in cancer progression and metastasis are influenced by Metadherin (MTDH), also known as AEG1 (Astrocyte Elevated Gene 1). MTDH protein expression in CRC tissues is considerably higher than that of normal tissues and may suppress the anti-cancer effects of miR-182-5p in CRC cells. Hence, the miR-182-5p/MTDH axis may be a promising target for CRC-targeted therapies [81].

5-Fluorouracil (5-FU) chemotherapy is a fundamental part of systemic chemotherapy for a wide range of malignancies, including CRC [82]. However, resistance to 5-FU is seen in approximately 90% of CRC patients. miR-182 has a high expression in CRC tissues and 5-FU-resistant CRC cell lines. Upregulation of miR-182 significantly induces drug resistance, proliferation, and colony formation, and causes apoptosis to be reduced in 5-FU-resistant CRC cell lines. miR-182 by direct targeting 3’-UTR of ST6GALNAC2 and consequent activation of PI3K/Akt pathway increases 5-FU resistance in CRC cells and causes CRC progression [83]. Sialyltransferases (STs) are pivotal enzymes associated with many biological processes, such as cellular recognition, cell signaling, cell-cell and host-pathogen interactions, and metastasis of cancer [84]. The improper expression of these enzymes has been confirmed in many cancers, including CRC. The expression level of ST6GALNAC2 as one of the STs is downregulated in CRC tissues and 5-FU-resistant CRC cell lines. Increased ST6GALNAC2 levels are associated with poor survival in CRC. Subsequently, miR-182 suppression and ST6GALNAC2-mediated inhibition of the oncogenic PI3K/Akt pathway increased chemosensitivity in 5-FU-resistant CRC cells [83].

At long last, according to studies, it can be claimed that miR-182 can play a potential role in the treatment of CRC by targeting multiple genes.

### Prostate cancer

Prostate cancer is a specific male cancer, and according to the latest global statistics published in 2018, with a nearly 1.3 million incidence rate and 359,000 deaths, it ranks fifth leading cause of cancer-related death in men. Both environmental and genetic factors, like other cancers, play a major role in prostate cancer progression. Prostate-specific antigen (PSA) blood test is one of the most important methods of prostate cancer screening, but the presence of defects, such as false positives, cause patients to face major problems. Prostate resection using surgery combined with chemotherapy and radiotherapy are common methods of treating this cancer, but the presence of drug resistance and the development of prostate cancer also make treatment difficult. By proving the role of microRNAs in metastasis and the development of prostate cancer, several groups of microRNAs that had altered expression in this cancer were identified; including miR-182, which was introduced as an important biomarker. Several studies on the expression of miR-182 in prostate cancer cells indicated that this microRNA acts as an oncomiR, and high expression of miR-182 in prostate cancer cells correlates with cell migration and invasion. During the investigations, the results illustrated an association between miR-182 overexpression and the suppression of FOXO1 tumor suppressor. FOXO1 belongs to the Forkhead box O transcription factor family and plays an important role in the cell cycle. Studies indicated that miR-182 is involved in increasing the invasion, migration, and cell proliferation of prostate cancer by targeting FOXO1 and thus suppressing the translation of this mRNA [85]. Another study reported that three genes, FOXF2, RECK, and MTSS1, which act as tumor suppressors and are involved in inhibiting the development of prostate cancer cells by reducing invasion and migration, were targeted by miR-182-5p. It should be noted that RECK shows its tumor suppressor function by reducing the invasion of prostate cancer by inhibiting MMP-2. However, FOXF2 shows the same function by inhibiting the expression of MMP-1 and increasing the inhibitor of MMPs expression, namely TIMP3. In connection with the role of FOXF2 in suppressing the invasive activity of prostate cancer cells, it was found that this tumor suppressor inhibits the activity of Wnt5a protein and Wnt signaling pathway. Inhibition of miR-182-5p by an inhibitor of this microRNA also resulted in the suppression of invasion and migration in prostate cancer cells via upregulation of FOXF2, RECK, and MTSS1 [86]. As mentioned earlier, Wnt/β-catenin signaling pathway is an important pathway for the development and progression of prostate cancer. Studies have shown that miR-182 contributes to the release of β-catenin from the degradation complex and its nuclear accumulation by targeting subtypes of the degradation complex, including APC, GSK-3β, Axin, and CK1. Following the process of β-catenin nuclear transfer, transcription of genes involved in the cell cycle with oncogenic functions, such as C-jun, C-myc, and CyclinD1, is upregulated [87].

Hypoxia is an important feature of cancer cells that mainly leads to angiogenesis. Hypoxia-inducible factor 1-alpha (HIF1α) has been identified as an important factor in prostate cell angiogenesis, which is controlled by HIF-prolyl hydroxylase (PHD) and the factor-inhibiting HIF (FIH) at protein and mRNA levels, respectively. The study showed that PHD and FIH are two direct targets of miR-182, and the inhibition of these two genes in the post-transcriptional phase is associated with increased HIF1α expression and angiogenesis in prostate cancer [88].

### Cervical cancer

Cervical cancer (CC) is one of the most common cancers specific to females, and every year a large number of women struggle with this problem. According to statistics, every year up to 530,000 women in the world suffer from this type of malignancy, nearly half of which, i.e. 260,000 patients die, making CC the fourth leading cause of cancer death in women. Despite preventive and therapeutic methods, such as vaccination, surgical resection, chemotherapy, and radiotherapy, which are part of the strategies to deal with CC, the treatment of advanced stages of this disease often fails and does not provide acceptable results. Studies on the mechanism of action of human papillomavirus (HPV) in cervical malignancy have shown that HPV oncogene, albeit an important etiologic factor in CC pathogenesis, is not able to cause malignancy in CC patients by itself, and host genetic variations are considered as key steps in the completion of this malignancy. Studies indicated that miR-182, depending on its target genes, can exhibit both tumor-suppressive and oncogenic properties. Studies of changes in the expression of miR-182 in the cervix have shown that the miR-182 level in cervical tumors is lower than that in normal tissue samples, indicating that miR-182 may act as an anti-cancer agent that reduces cell proliferation and promotes apoptosis in this cancer [89–92]. Further studies have shown that miR-182 has an inhibitory effect on DNMT3a, a gene known as a DNA (cytosine-5)-methyltransferase. By reducing the expression of miR-182, DNMT3a expression can be upregulated, resulting in hypermethylation of tumor suppressors and inhibition of apoptosis [89].

As mentioned earlier, HPV promotes CC progression, and research has shown that E7 and E6 oncogenes expressed by this virus play an undeniable role in this process by suppressing pRb and p53 tumor suppressors, respectively. Further studies reported an increase in miR-182 expression in CC with the involvement of high-risk HPV E7. E7 protein targets pRb and leads to the release of E2F from pRb. By nuclear translocation of E2F and binding to the TGF-β gene promoter, E2F increases the expression of TGF-β gene. TGF-β secretion to the tumor environment induces TGF-β / Smad4 signaling pathway. Finally, it was found that Smad4 binds to the miR-182 promoter and increases the expression of this microRNA, which is associated with CC progression [91]. Attention to the role of lncRNAs in the progression and even suppression of tumors has increased in recent years. Research on their role in CC suggests that lncRNAs are involved in both the progression and suppression of CC. One study showed that lncRNA PCGEM1 could suppress and sponge miR-182 to upregulate an oncogenic member of the F-box protein family, called FBXW11, leading to CC cell proliferation, invasion, and migration. miR-182-mediated FBXW11 inhibition was able to inactivate NF-κB and B-catenin / TCF signaling pathway, which was reversible by PCGEM1 overexpression [90]. In another study focusing on the association between lncRNAs and miR-182 in CC progression, it was found that LINC00173 lncRNA exhibits a tumor suppressor role and induces the expression of another member of the F-box protein family, FBXW7, by sponging miR-182. FBXW7 is considered a tumor suppressor that diminishes CC cell proliferation and invasion. However, miR-182 was found to increase tumor progression by targeting FBXW7. LNC00173 overexpression, therefore, could rescue FBWX7 expression, suppressing CC cell proliferation and invasion [92].

### Osteosarcoma

Osteosarcoma (OS) is a type of malignancy that mainly affects adolescents and associates with primary bone tumors. Studies of changes in miR-182 expression have shown that this microRNA is also downregulated in OS. One of these studies points out the interaction between miR-182 and HOXA9 (Homeobox A9), which is known as a transcription factor involved in fetal development. miR-182 regulates Wnt / β-catenin signaling pathway by targeting HOXA9. Following the downregulation of miR-182, Wnt / β-catenin signaling pathway is activated to increase cell proliferation and inhibit apoptosis in OS cells. Another direct target of miR-182 is a member of the guanine nucleotide exchange factor family (GEFs), called TIAM1. TIAM Rac1 Associated GEF 1 (TIAM1) is overexpressed in various cancers, such as breast, lung, and gastric cancers. Since TIAM1 is a direct target of miR-182, following the downregulation of miR-182, TIAM1 is also overexpressed and subsequently increases cell proliferation, migration, and metastasis in OS cells [93, 94]. The studies that have investigated the causes of OS malignancy have shown that invasion and migration of OS can be due to increased expression levels of EBF2, a member of the COE (Collier/Olf/EB) family. EBF2 is a direct target of miR-182, which is overexpressed as a result of miR-182 downregulation in OS, showing an association with increased expression of osteoprotegerin (OPG), tumor necrosis factor receptor superfamily member 11B. Subsequently, the rescue of EBF2 from miR-182 resulted in the suppression of TRAIL-induced apoptosis and the induction of cell migration and invasion [95].

### Breast cancer

Around the world, breast cancer (BC) is the foremost common type of malignant tumors as well as the major cause of cancer-related death in women [96]. BC is considered a heterogeneous disease, and the expression and amplification of human epidermal growth factor receptor type 2 (HER2), and the absence or presence of estrogen receptor (ER) and progesterone receptor (PR), cause this disease to be divided into three clinical subgroups; HER2-positive (HER2+), triple-negative (TN; ER–, PR–and HER2–), hormone-receptor (HR)-positive (HR+; ER+, PR+/– and HER2–) [97]. The expanding prevalence of BC is associated with several physiological abnormalities. Analysis of microRNA-related targets and signaling pathways involved in BC showed that miR-182 is significantly upregulated in BC cells and tissue or serum samples of BC patients. According to numerous pieces of evidence, miR-182 has been introduced as a new and valuable biomarker for the diagnosis and treatment of BC [98, 99].

During the evaluation of the miR-183/182/96 cluster expression in several tumors, it was shown that the miR-183/182/96 cluster expression level was upregulated in BC cell lines and tissues in comparison to normal cell lines and adjacent normal tissues, respectively. Also, this upregulation was associated with poor pathological clinical parameters and, of course, poor prognosis of patients. The results indicated that the miR-183/182/96 cluster is a new prognostic biomarker for BC [100].

When issues, such as cell cycle progression, protecting of DNA integrity, apoptosis, regulation of a collection of specific transcriptional pathways, and maintenance of telomere length, arise, the role of BRCA1 and BRCA2 genes belonging to the family of ATM-mediated DNA double-strand breaks (DSBs) repair genes, is becoming more prominent [101, 102]. Germline mutations or deletions in BRCA1 and BRCA2 genes are associated with breast tumor formation [103]. Decreased BRCA1 expression in sporadic breast tumors is associated with disease progression and poor prognosis. The suppression of miR-182 was found to be associated with BRCA1 protein upregulation, DNA repair, and the resistance of breast tumor cells to inhibitors of poly (ADP-ribose) polymerase1 (PARP1) and irradiation-induced cell death [104]. This finding indicates the controversial function of miR-182 dysregulation through breast tumorigenesis as well.

Besides, studies have reported the relationship between miR-182 and Wnt/β-catenin signaling. It has been shown that β-catenin, which plays an important role in signal transduction, cell adhesion, and regulation of gene expression, can be positively regulated by miR-182, leading to breast tumorigenesis. On the other hand, it was detailed that RECK, as an MMP inhibitor, is inversely regulated by miR-182. Therefore, it is noteworthy that miR-182 overexpression resulted in cell invasion and colony formation in BC cells by targeting RECK [105].

In another study, CircRNA_000554 downregulation has been reported to be correlated with high expression of miR-182 in BC tissues. It was shown that increasing CircRNA_000554 could suppress migration, invasion, and EMT by sponging miR-182 and rescuing ZFP36 expression. Downregulated miR-182, moreover, suppressed TGFβ and hampered EMT by diminishing the expression of Twist1, Vimentin, and N-cadherin, and increasing the expression of E-cadherin. On another side, the silencing of miR-182 could suppress BC cell invasion and cell proliferation by upregulating ZFP36 [96]. Zinc finger protein (ZFP36), as an AU-rich element-binding protein, plays a part in post-transcriptional gene regulation by degrading target mRNAs [106]. ZFP36 was found to be a direct target of miR-182 and can inhibit EMT by inhibiting Snail1 and Twist1 in cancer cells [96]. Also, ZFP36 has a suppressive effect on cell migration and proliferation by inhibiting Wnt/β-catenin signaling pathway [98]. Besides, the circRNA_000554/miR-182/ZFP36 axis was evidenced to participate in regulating cell cycle progression, cellular apoptosis, and autophagy in BC cells [96].

Another study reported the oncogenic effect of miR-182-5p by targeting CASP9 in MCF-7 BC cells. In this regard, with the introduction of LNA-anti-miR-182-5p to BC cells, the expression of miR-182-5p was diminished, leading to CASP9 overexpression. As a result, BC cell survival and proliferation were reduced while cell apoptosis was induced [107]. Locked nucleic acid (LNA) is known as a class of nucleic acid analogs, which possess very high affinity and excellent specificity toward complementary DNA and RNA. Owing to its enzymatic resistance features, nontoxicity, stability, and nonproduction of the immune response, LNA is considered a promising option for gene therapies [107, 108].

Also, miR-182 has been found to regulate FOXO1 mRNA and protein levels in MCF-7 BC cells by targeting 3’-UTR of FOXO1. Decreased FOXO1 mRNA level was observed in breast tumor specimens compared to normal breast tissues. The transcription factor FOXO1 is a tumor suppressor that regulates cell cycle progression, cellular metabolism, and apoptosis induction. It was reported that miR-182 suppression led to a significant increase in FOXO1 expression, inhibiting cell cycle progression, cell proliferation, and colony formation and inducing apoptosis in BC cells [109].

Studies have also indicated the function of miR-182 in Triple-Negative Breast Cancer (TNBC). The expression of miR-182 has been evidenced to be correlated with clinical features of TNBC, such as intravascular cancer emboli, lymph node metastasis, TNM (stage III), and TNBC recurrence. miR-182 was also significantly higher in MDA-MB-231 (TNBC) cells than that in MCF7 (ER^+^) cells. Overexpression of miR-182 in vitro increases BC cell proliferation, invasion, and cell migration [110, 111] and leads to increased tumor volume and metastasis within the lungs of mouse models of BC [111]. Reports indicated that miR-182 can play an imperative part in the progression and development of TNBC by targeting FOXF2 [110, 111]. The mesenchymal regulator FOXF2 belongs to the FOX transcription factor superfamily and plays an important role in tissue homeostasis by regulating EMT to maintain epithelium polarity [112]. Besides, it has been evidenced that insufficient expression of FOXF2 is related to poor prognosis and early metastasis of patients with TNBC [110, 111].

Increased expression of miR-182-5p in BC cells and tissues is associated with poor survival, proliferation, and invasion. The occurrence of a variety of biological results is the outcome of the function of a tumor suppressor, called phosphatase and tensin homolog deleted on chromosome 10 (PTEN), which is involved in regulating metabolism and the growth process in BC cells. It was shown that PTEN is a direct target of miR-182-5p. Consequently, miR-182-5p suppression leads to PTEN overexpression, diminishes BC cell invasion and proliferation, and hampers tumor growth in the murine xenograft models [113].

Targeted drug, trastuzumab, is a humanized monoclonal antibody that, by affecting signaling pathways, leads to antibody-dependent cellular toxicity and the HER2 receptor degradation, induction of apoptosis, and anti-angiogenic effects. The human epidermal growth factor receptor 2 (HER2) is a receptor tyrosine-protein kinase encoded by ERBB. Overexpression of HER2 by controlling the PI3K/Akt and Ras/Raf/MEK/MAPK pathways leads to early systemic metastasis, migration, differentiation, and proliferation of malignant cells [114, 115]. miR-182 modulates the resistance of BC cells, including SKBR3/TR and BT474/TR, to trastuzumab by targeting MET protooncogene and regulating the signaling pathway PI3K/Akt/mTOR. So, the miR-182 mimic by inhibiting MET/PI3K/Akt/mTOR axis leads to sensitization of trastuzumab-resistant cells to trastuzumab and reduces cell migration and invasion. In contrast, miR-182 knockdown and activation of MET/PI3K/Akt/mTOR axis lead to increased trastuzumab-resistant in trastuzumab-resistant cells and induced invasion and migration [115].

Altogether, these studies on the miR-182 and target genes in BC have suggested potential strategies for improving the treatment of this malignancy.

### Glioma

Glioma, which accounts for 30% of all central nervous system and brain tumors, is the most common primary brain tumor. Glioma is divided into two main subgroups: diffuse glioma and non-diffuse glioma. Therefore, diffuse glioma is divided into 4 grades (I-IV) according to WHO classification and in terms of malignancy severity, grade IV with the highest severity of malignancy is known as glioblastoma [116–119]. Studies indicated the dual role of miR-182 in suppressing as well as promoting glioblastoma. It has been shown that overexpression of miR-182 by interacting with 3’UTR of FBXW7, a gene involved in the ubiquitination of oncoproteins, suppressed the translation of this anti-tumor gene and reduced its expression. miR-182-mediated FBXW7 downregulation resulted in the inhibition of apoptosis, followed by increased cell proliferation and migration in glioma cells [117]. According to a report, another target gene for miR-182 includes a member of the cadherin family, called PCDH8, which acts as a tumor suppressor and is downregulated by overexpression of miR-182 in glioma cells. STAT3 / miR-182 / PCDH8 axis plays a key role in the progression of this type of cancer. STAT3 activity induces transcription of the miR-182-5p gene by binding to the promoter region of this microRNA. miR-182-5p overexpression, in turn, increases the rate of migration, invasion, and cell proliferation by targeting PCDH8 [120].

According to studies performed on the U251 cell line, miR-182 downregulation participates in the regulation of cell proliferation and invasion, so the transfection of miR-182 to these cells reduced the invasion. Neuritin protein, which is known as a member of the family of neurotropic factors, plays a key role in the neurite outgrowth and the synaptic maturation of the central nervous system. It has shown that miR-182 diminishes cell proliferation and invasion in glioma by targeting the gene encoding Neuritin protein, NRN1 [121]. Further studies found an interaction between a type of lncRNA and miR-182, which results in increased glioma cell proliferation. This lncRNA, called UCA1 (urothelial carcinoma associated 1), modulates the expression of iASPP (inhibitor of apoptosis-stimulating protein of p53) by interacting with miR-182 and induces tumorigenesis. iASPP is reported to be a direct target of miR-182 [118]. Another study focused on the tumor suppressive effect of miR-182 in glioblastoma showed that miR-182 overexpression diminished in vivo and in vitro glioblastoma tumorigenesis by targeting Protein phosphatase 1 regulatory inhibitor subunit 1 C (PPP1R1C). PPP1R1C acts as an oncogene in glioblastoma, the high expression of which was shown to participate in cell proliferation, migration, and invasion and the Temozolomide (TMZ) chemosensitivity of glioblastoma patients. Consequently, miR-182 overexpression was able to inhibit PPP1R1C activity and increase the sensitivity of GBM cancer cells to TMZ [122].

### Pancreatic cancer

**Fig. 4:**
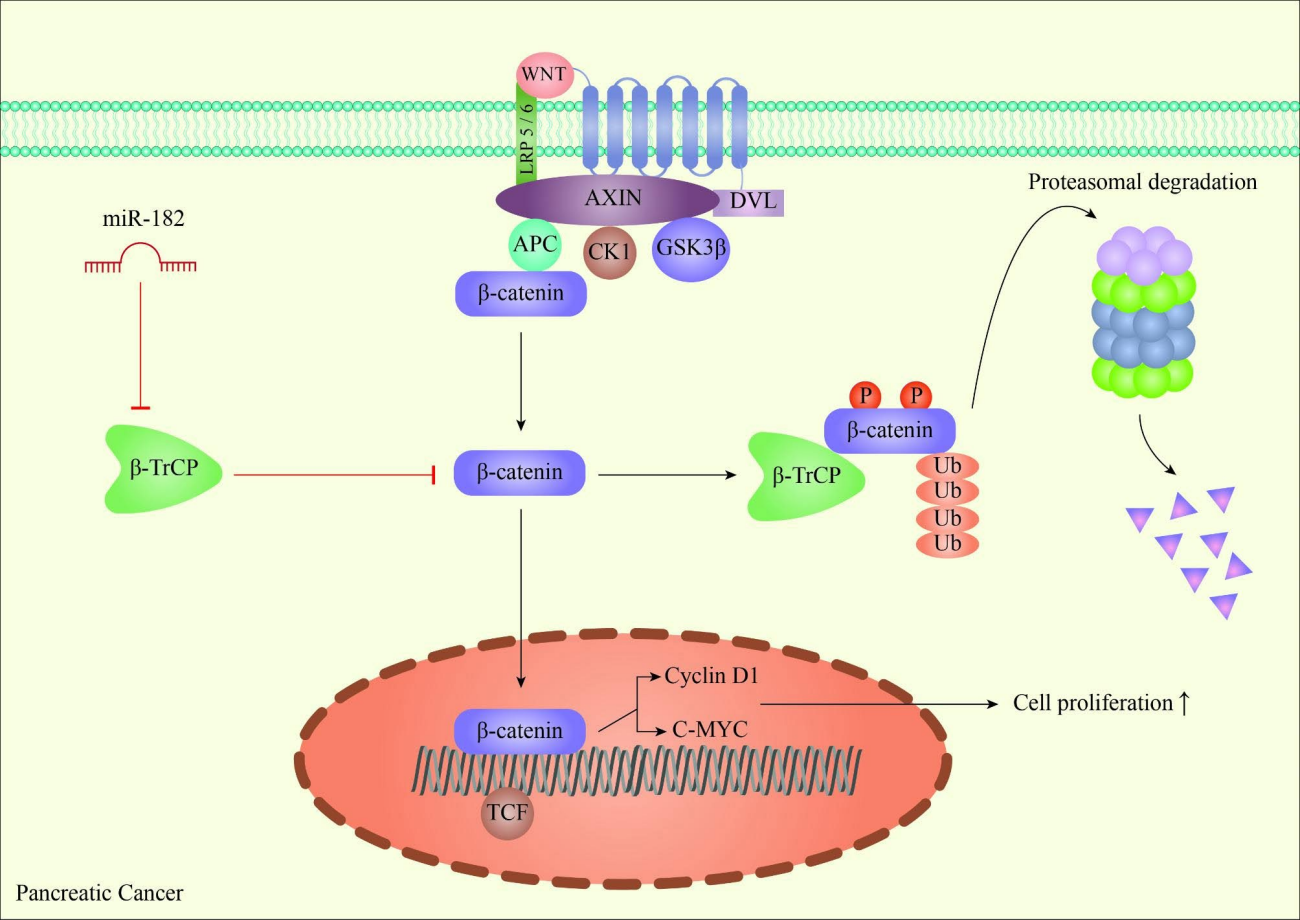
The schematic view of miR-182 involvement in β-catenin signaling through pancreatic tumorigenesis. β-TrCP, which induces the proteasome-mediated degradation of β-catenin, is inhibited by miR-182, promoting cell proliferation by β-catenin-mediated upregulation of C-myc and Cyclin D1

Pancreatic cancer, as a highly invasive malignancy, ranks fourth in terms of cancer mortality in Western countries, and due to the late diagnosis of this disease, the 5-year survival rate reaches only about 5%. Studies on the central role of miR-182 indicated the overexpression of this microRNA in pancreatic cancer, which resulted in cell proliferation and invasion. β-TrCP2, as an interactor with phosphorylated substrates, is a member of the large F-box family that recruits SCF^β−TrCP^ E3 ubiquitin ligases to its target. These ligases prevent malignancy by proteasomal degradation of components involved in tumor-promoting signaling pathways, such as β-catenin. It has been shown that β-TrCP2 is a direct target of miR-182, and it is downregulated in pancreatic cancer, leading to the overexpression of β-catenin and downstream components of β-catenin / TCF signaling pathways, such as C-myc and cyclin D1(Fig. 4) [120]. These findings indicate the oncogenic role of miR-182 in pancreatic cancer progression.

### Melanoma

Melanoma is one of the cancers that is known with highly aggressive and metastatic properties, and the only effective treatment is resection surgery of primary melanoma, indicating the need for identifying novel therapeutic targets and strategies. Research into the effects of miR-182 dysregulation in melanoma has evidenced that miR-182 plays a dual role in this malignancy as well. Miguel F Segura and colleagues illustrated that miR-182 is overexpressed in melanoma cell lines and tissue samples, participating in increased survival and migration of melanoma cells in vitro and in vivo. The results indicated that two genes, including FOXO3 and Microphthalmia-associated transcription factor-M (MITF-M), are targeted by miR-182 in this process. MITF is deemed to be an important differentiation factor and is well known for its role in suppressing cell proliferation, especially by activating cell cycle inhibitors including p16^INK4a^ and p21^Cip1^ as well as controlling cell migration by regulating DIA. Opposite to these findings, in posterior uveal melanoma, miR-182 has been found to function as a suppressor tumor that suppresses the expression of MITF, Bcl-2, and cyclin D2. MITF directly regulates the activity of c-Met oncogene, which in turn activates AKT and ERK1/2 pathways. Subsequently, p53-mediated induction miR-182 expression reduced melanoma cell migration and proliferation by regulating MITF and thus reducing c-Met expression [123–125]. In another study conducted by Jiayue Ding and colleagues on melanoma, it was found that miR-182 was overexpressed and acted as an oncogene in this malignancy. On the other hand, RECK, which is a negative regulator of metastasis, was downregulated. In this study, the luciferase reporter assay showed that RECK is a direct target of miR-182, and its expression is suppressed by increasing the expression of miR-182 in melanoma cells. Subsequently, it was evidenced that RECK overexpression mediated by miR-182 silencing could prevent melanoma cancer cell proliferation and progression [126, 127].

### Lung cancer

Based on studies, if we want to point out the main cause of cancer-caused mortality, lung cancer (LC) will be ranked first [3]. Non-small-cell lung cancer (NSCLC, 85% of LC cases) and small-cell lung cancer (SCLC, 15% of LC cases) are the two main groups of lung cancer classified on a pathological basis. NSCLC is the main sort of LC that can be further classified into adenocarcinomas, squamous cell carcinomas, and large cell carcinomas [128]. Despite different treatment methodologies, the prognosis and diagnosis of patients with LC are poor, and the five-year survival rate is less than 18% [129].

Increased expression of miR-182 in NSCLC tissues compared to adjacent normal tissues is correlated with malignant phenotypes in NSCLC. miR-182 promote cell growth, cell cycle progression, and colony formation by directly targeting and repressing the expression of FBXW11 and FBXW7. FBXW11 and FBXW7 are two important F-box proteins of the ubiquitin-proteasome system (UPS) that have been recognized as tumor suppressors, by targeting oncogenic proteins, in a different variety of human cancers. Downregulation of miR-182 or enhancing FBXW11/FBXW7 may be a valuable therapeutic methodology for NSCLC treatment [26].

Studies have shown that PDCD4 is also considered a direct target of miR-182 in lung cancer, indicating the oncogenic function of this microRNA [130]. Programmed cell death (PDCD4), known as a tumor suppressor, has functions, such as modulating signaling pathways, diminishing tumorigenesis, and influencing the translation and transcription process of multiple genes [130, 131]. It has been reported that miR-182 downregulation is directly associated with increased PDCD4 expression and causes cell proliferation and metastasis and invasion of human lung adenocarcinoma cells to be suppressed [130]. Also, due to the fundamental role of miR-182 in the development of drug resistance, it has been detailed that by reducing the expression of miR-182 and increasing PDCD4 expression, the NSCLC cell chemoresistance against cisplatin was hindered [132].

Besides, increased expression of miR-182 has been identified as a factor involved in the radioresistance of NSCLC. It was shown that miR-182 suppression leads to decreased cell survival, increased cell apoptosis, cell cycle arrest, and increased DNA damage [133]. This was achieved by targeting FOXO3. This protein plays an important role in controlling cell growth and apoptosis [134] and is also involved in responding to DNA damage to maintain genome stability. Subsequently, targeting miR-182 / FOXO3 axis was found to be effective in radiosensitizing NSCLC cells by affecting the repair of DNA damage [133].

In opposition to previous studies, a study has indicated that miR-182 can act as a tumor suppressor in LC by regulating the expression of a member of the G protein signaling family regulator encoded by the RGS17 gene. Increased expression of RGS17 in human lung adenocarcinomas plays an important role in cell proliferation and growth. miR-182 was found to target and suppress RGS17 and thus prevent colony formation, proliferation, and growth in LC cells. Decreased expression of RGS17, in addition to inhibiting NSCLC cell proliferation in vitro, diminished in vivo tumor progression [128].

**Fig. 5:**
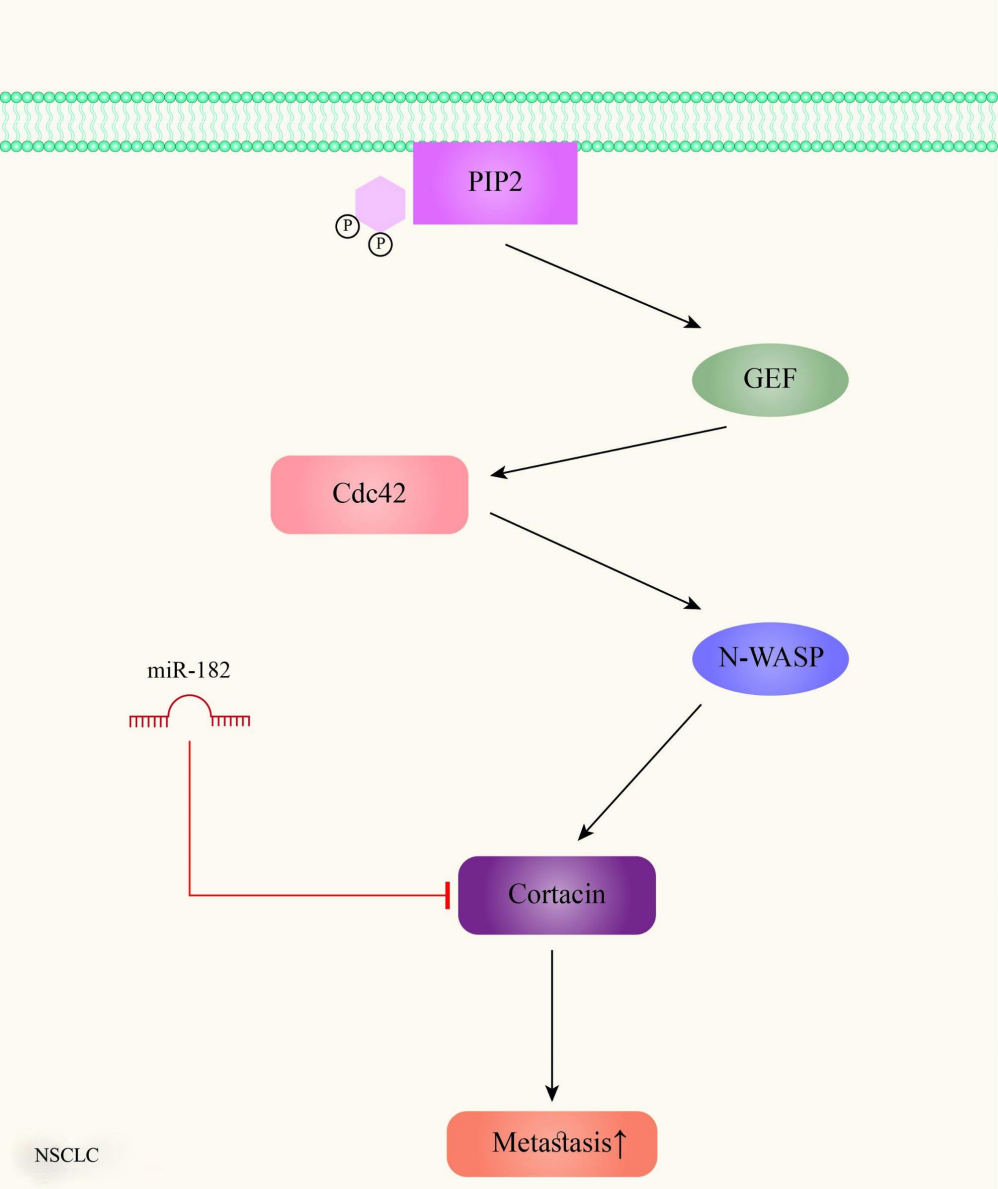
The involvement of miR-182 in lung cancer metastasis. Cortactin oncogene, a mediator of the Cdc42/N-WASP pathway inducing cancer cell invasion, can be inhibited by miR-182 tumor suppressor

Cortactin, a fundamental protein involved in tumor cell invasion, is frequently overexpressed in tumors and is associated with poor prognosis and metastasis in patients. Cortactin, as a marker of invadopodia, plays an imperative role in their function. Furthermore, it should be noted that cortactin is responsible for the regulation of matrix metalloproteinase secretion and ECM debasement in invadopodia. The formation of metastatic cascades following the destruction of the basement membrane and the surrounding stroma is one of the important roles of these actin-rich plasma membrane protrusions called invadopodia. miR-182 is reported to have the ability to suppress cancer cell metastasis, by suppressing the Cdc42/N-WASP pathway as well as the phorbol 12,13-dibutyrate (PDBu), HGF, Rac1, and Rock1 activities through targeting cortactin (Fig. 5). Thus, miR-182 suppresses invadopodia formation and ECM degradation and subsequently suppresses NSCLC cell metastasis by directly targeting cortactin [135].

Another study showing the oncogenic role of miR-182 has established that miR-182-5p is upregulated in serum and tissue samples of NSCLC patients, and its overexpression could considerably suppress AGER mRNA and protein expression. Subsequently, NF-κB pathway activity mediated by AGER suppression promoted in vitro cell proliferation, invasion, and metastasis. On the other hand, it was shown that LINC00173, as a ceRNA, negatively regulates miR-182-5p in NSCLC and modulates the miR-182-5p/AGER/NF-κB axis. The overexpression of LINC00173 by negatively regulating miR-182-5p was able to diminish NSCLC cell malignant features by upregulating AGER [129].

Specificity protein 1 (Sp1)-mediated overexpression of miR-182 has been also reported to contribute to lung cancer progression. Sp1 transcription factor, a member of a specificity protein / Krüppel-like family, positively regulates miR-182 expression, which in turn inhibits FOXO3 translational activity and subsequently promotes the growth of cancer cells n the early stages of lung cancer progression. However, Sp1 downregulation in the late stages of lung cancer leads to a decrease in miR-182 expression, causing FOXO3-mediated tumor metastasis by increasing the expression of N-cadherin, ADAM9, CDH9, and CD44 [136].

All in all, it can be suggested that targeting miR-182 may be a promising target for NSCLC treatment in different stages of malignancy.

### Esophageal squamous cell

According to global statistics, esophageal cancer ranks sixth in cancer-related mortality. The main reason for this high mortality rate is the asymptotic progression of this cancer and the lack of early diagnosis. Esophageal squamous cell (ESCC) is one of the main pathological types of esophageal cancer associated with invasion and high cell proliferation in patients. Research on the role of miR-182 in esophageal cancer suggested that this microRNA is upregulated in ESCC, and its suppression could decrease cell proliferation and invasion and promote cell apoptosis and cell cycle arrest in G0 / G1 phase. YWHAG, which functions as a tumor suppressor in various cancers and encodes 14-3-3 protein gamma, was identified as the downstream target of miR-182. Suppression of YWHAG by upregulating miR-182 resulted in increased cell proliferation and metastasis with increased cancer cell invasion [137], indicating miR-182 as a promising target for ESCC.

### Renal cell carcinoma

**Fig. 6:**
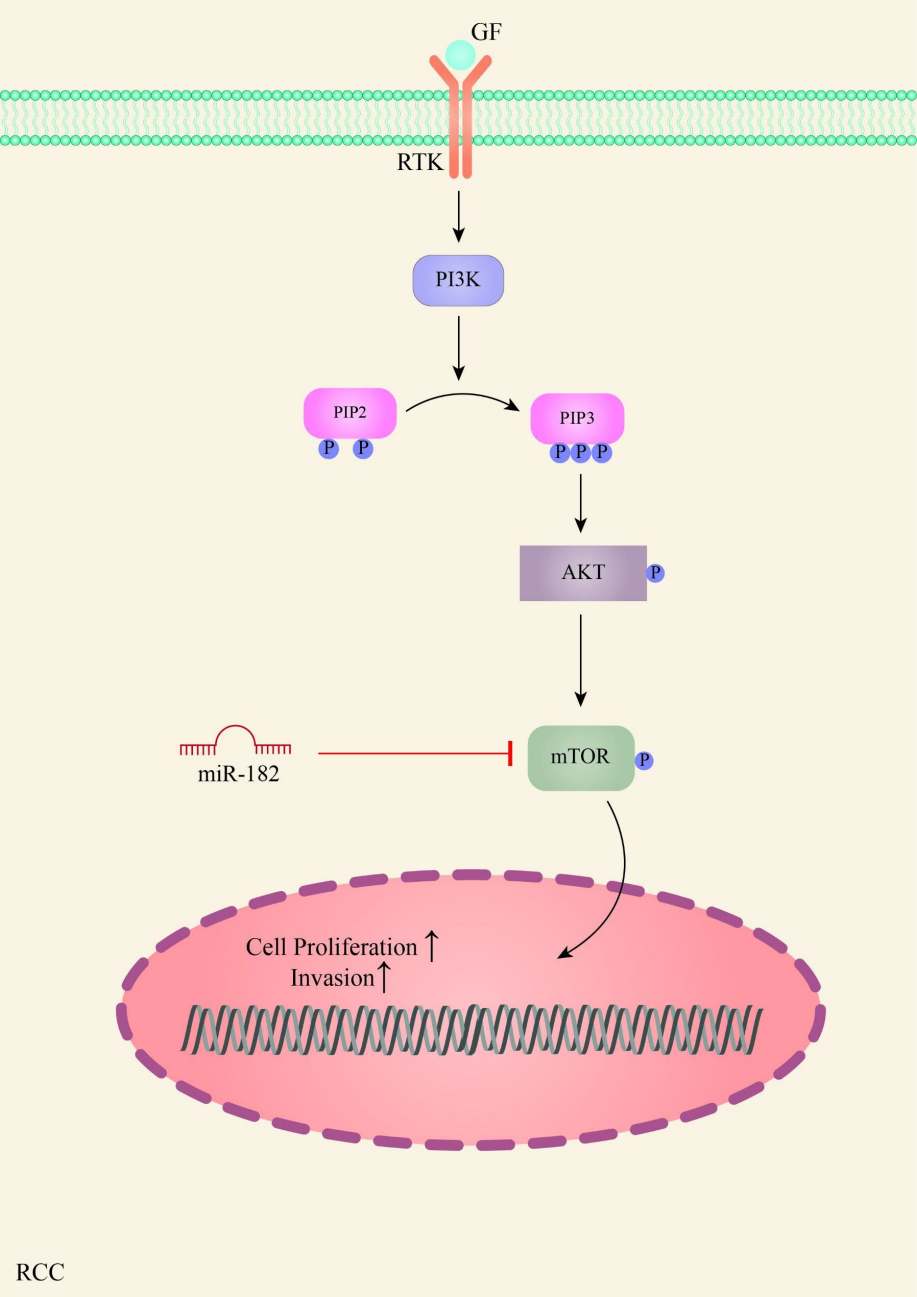
A schematic view of the regulation of the P13K/AKT/mTOR signaling in RCC by miR-182. mTOR, a downstream component of this pathway, is directly targeted by miR-182, suppressing cell proliferation and invasion

The most common type of cancer that affects the kidneys is known as Renal Cell Carcinoma (RCC) and clear cell RCC (ccRCC) is the most common histological type of this kind of cancer. In vitro and in vivo tests indicated the tumor suppressive role of miR-182 in RCC. This microRNA is downregulated in RCC and regulates the expression of FLOT1 (Flotillin 1). This protein function as a molecular mediator that connects membrane receptors and signal pathways, playing an important role in regulating the AKT / FOXO3a signaling pathway. FLOT1 was shown to be a direct target of miR-182, and its suppression mediated by miR-182 could reduce cell proliferation and tumorigenesis by inactivating the AKT / FOXO3a pathway [138]. Other studies into the role of miR-182 in RCC suggested that IGF1R is another target of miR-182. IGF1R is known as a very important oncogene that mediates cell survival, migration, and proliferation. Subsequently, its overexpression following miR-182 suppression has been reported to contribute to RCC progression [139]. Another signaling pathway that is regulated by miR-182 in RCC tumorigenesis includes P13K / AKT / mTOR pathway. In this regard, mTOR has been found to be a direct target of miR-182. mTOR, as a downstream gene of the P13K / AKT / mTOR signaling pathway, is phosphorylated following AKT phosphorylation, leading to increased cell invasion and proliferation. Consequently, overexpression of miR-182 was evidenced to inhibit mTOR signaling and prevent cell proliferation and invasion in RCC (Fig. 6) [140].

One of the main causes of the downregulation of tumor suppressor microRNAs, including miR-182, through RCC tumorigenesis, is hypermethylation. It has been shown that Aza-CdR treatment leads to the downregulation of DNMT1, DNMT3a, and DNMT3b DNA methyltransferases in ccRCC cells and increases miR-182. In turn, miR-182 overexpression was shown to target MALAT1 lncRNA and inhibit ccRCC proliferation while inducing cell cycle arrest at the G2 / M phase. This was achieved by the consequent upregulation of p53, and downregulation of CDC20 and AURKA, drivers of the cell-cycle mitotic phase [141].

Table 1 summarizes the studies that investigated the role of miR-182 in different types of human cancers.

**Table 1:** The functions and targets of miR-182 in various human cancers

Cancer	Expression	Target Gene	Function	Pathway	Reference
**Gsatric cancer**	Upregulation Downregulation Downregulation Downregulation Downregulation Downregulation Downregulation	RAB27A CREB1 ANUBL1 UCA1/TIMP2 HOXA9/RUNX3 circFN1 circ-SFMBT2/ CREB1	Increased Invasion, Migration and Proliferation Decreased cell proliferation Decreased Colony formation and Proliferation Decreased Invasion and Migration Decreased Proliferation and Invasion, Migration Induction of apoptosis Decreased Proliferation and induction of apoptosis	PI3K/Akt/GSK3β, NF-κB	33 32 39 40 44 50 51
**Hepatocellular carcinoma**	Upregulation Upregulation Upregulation Upregulation Upregulation Upregulation	MTSS1 FOXO3a FOXO1 PDCD4 Ephrin5A RASA1	Increased metastasis Increased proliferation Increased proliferation Tumorigenesis Increased proliferation Angiogenesis	Wnt/B-catenin	56 55 58 59 60 61
**Colorectal cancer**	Upregulation Upregulation Upregulation Upregulation Upregulation Downregulation Upregulation	Lnc-AGER-1, AGER lncRNA GAS5, GAS5, FOXO3a SATB2 DAB2IP FoxF2 MTDH ST6GALNAC2	Increased Proliferation and Migration Increased Proliferation Increased metastasis Increased Proliferation, Invasion and Angiogenesis Invasion, Proliferation and Metastasis Decreased Proliferation, Invasion and Metastasis Increased Drug resistance, Proliferation, Colony formation	NF-κB and Erk1/2, PI3K /Akt Wnt/β-catenin, PI3K/Akt/mTOR Wnt/β-catenin PI3K/Akt	71 72 75 77 79 80 82
**Prostate cancer**	Upregulation Upregulation Upregulation Upregulation	FOXO1 RECK FOXF2 PHD, FIH	Increased invasion, migration and proliferation Increased invasion Increased invasion Angiogenesis	RECK/MMP2 Wnt	84 85 85 87
**Cervical cancer**	Downregulation Upregulation	DNMT3a FBXW7	Hypermethylation of tumor suppressor genes Increased invasion and cell proliferation		88 91
**Osteocarcinoma**	Downregulation Downregulation Downregulation	HOXA9 TIAM1 EBF2	Decreased cell proliferation and induction of apoptosis Decreased cell proliferation, metastasis and migration Decreased invasion and migration	Wnt/B-catenin	92 93 94
**Breast cancer**	Upregulation Upregulation Upregulation Upregulation Upregulation Upregulation Upregulation	BRCA1, BRCA2 RECK circRNA_000554, ZFP36 CASP9 FOXO1 FOXF2 PTEN	Increased Resistance to PARP1 Increased Invasion, Colony formation Increased Migration, Invasion and Proliferation Increased Survival, proliferation Increased Proliferation and Colony formation Increased Metastasis Increased Proliferation and invasion	Wnt/β-catenin TGFβ/Vimentin/ N-cad/E-cad/Snail1/ Twist1	102 104 95 106 108 109, 110 112
**Glioma**	Upregulation Upregulation	FBXW7 PCDH8	Increased cell proliferation and migration Increased invasion and migration	JAK/STAT3	116 119
**Pancreatic cancer**	Upregulation	β-TrCP2	Tumor progression	β-catenin / TCF	121
**Melanoma**	Upregulation	MITF-M	Increased cell proliferation and migration		123
**Lung cancer**	Upregulation Upregulation Upregulation Downregulation Downregulation Upregulation Upregulation	FBXW11, FBXW7 PDCD4 FOXO3 RGS17 Cortactin LINC00173, AGER Sp1, FOXO3	Increased cell growth and Colony formation Increased Proliferation, metastasis and invasion Decreased Radiosensitizing Decreased Colony formation, Proliferation and cell Growth Decreased Metastasis Increased Proliferation and Migration Increased Invasion and Migration	Cdc42/N-WASP NF-κB N-cadherin, ADAM9	23 127, 129 130 125 132 126 133
**Esophageal squamous**	Upregulation	YWHAG	Increased cell proliferation and metastasis		134
**Renal cell carcinoma**	Downregulation Downregulation Downregulation	FLOT1 IGF1R mTOR	Decreased cell proliferation Mediated migration and cell proliferation Decreased cell proliferation and invasion	Akt/FOXO3a P13k/Akt/mTOR	135 136 137

## Conclusion

A growing number of studies have indicated that one of the main mechanisms participating in the initiation and progression of a wide range of human cancers is the dysregulation of microRNAs, which play a key role in regulating biological processes. As discussed in this study, miR-182 is one of the well-studied microRNAs, which regulates multiple target genes and signaling pathways and plays a dual role in various malignancies. Subsequently, miR-182 dysregulation participates in regulating different aspects of in vivo and in vitro tumorigenesis, such as apoptosis, metastasis, proliferation, invasion, and drug resistance. These findings, altogether, imply that miR-182 can be a promising target in the treatment and diagnosis of various malignancies, demanding further investigations toward clinical applications.

## Data Availability

Not applicable.
